# Large language models for error detection in radiology reports: a comparative analysis between closed-source and privacy-compliant open-source models

**DOI:** 10.1007/s00330-025-11438-y

**Published:** 2025-02-20

**Authors:** Babak Salam, Claire Stüwe, Sebastian Nowak, Alois M. Sprinkart, Maike Theis, Dmitrij Kravchenko, Narine Mesropyan, Tatjana Dell, Christoph Endler, Claus C. Pieper, Daniel L. Kuetting, Julian A. Luetkens, Alexander Isaak

**Affiliations:** 1 Department of Diagnostic and Interventional Radiology, University Hospital Bonn, Bonn, Germany; 2 Quantitative Imaging Lab Bonn (QILaB), University Hospital Bonn, Bonn, Germany

**Keywords:** Generative pre-trained transformers, Large language models, Open source, Error detection, Radiology reports

## Abstract

**Purpose:**

Large language models (LLMs) like Generative Pre-trained Transformer 4 (GPT-4) can assist in detecting errors in radiology reports, but privacy concerns limit their clinical applicability. This study compares closed-source and privacy-compliant open-source LLMs for detecting common errors in radiology reports.

**Materials and methods:**

A total of 120 radiology reports were compiled (30 each from X-ray, ultrasound, CT, and MRI). Subsequently, 397 errors from five categories (typographical, numerical, findings-impression discrepancies, omission/insertion, interpretation) were inserted into 100 of these reports; 20 reports were left unchanged. Two open-source models (Llama 3-70b, Mixtral 8x22b) and two commercial closed-source (GPT-4, GPT-4o) were tasked with error detection using identical prompts. The Kruskall–Wallis test and paired *t*-test were used for statistical analysis.

**Results:**

Open-source LLMs required less processing time per radiology report than closed-source LLMs (6 ± 2 s vs. 13 ± 4 s; *p* < 0.001). Closed-source LLMs achieved higher error detection rates than open-source LLMs (GPT-4o: 88% [348/397; 95% CI: 86, 92], GPT-4: 83% [328/397; 95% CI: 80, 87], Llama 3-70b: 79% [311/397; 95% CI: 76, 83], Mixtral 8x22b: 73% [288/397; 95% CI: 68, 77]; *p* < 0.001). Numerical errors (88% [67/76; 95% CI: 82, 93]) were detected significantly more often than typographical errors (75% [65/86; 95% CI: 68, 82]; *p* = 0.02), discrepancies between findings and impression (73% [73/101; 95% CI: 67, 80]; *p* < 0.01), and interpretation errors (70% [50/71; 95% CI: 62, 78]; *p* = 0.001).

**Conclusion:**

Open-source LLMs demonstrated effective error detection, albeit with comparatively lower accuracy than commercial closed-source models, and have potential for clinical applications when deployed via privacy-compliant local hosting solutions.

**Key Points:**

***Question*** *Can privacy-compliant open-source large language models (LLMs) match the error-detection performance of commercial non-privacy-compliant closed-source models in radiology reports?*

***Findings*** *Closed-source LLMs achieved slightly higher accuracy in detecting radiology report errors than open-source models, with Llama 3-70b yielding the best results among the open-source models.*

***Clinical relevance*** *Open-source LLMs offer a privacy-compliant alternative for automated error detection in radiology reports, improving clinical workflow efficiency while ensuring patient data confidentiality. Further refinement could enhance their accuracy, contributing to better diagnosis and patient care.*

**Graphical Abstract:**

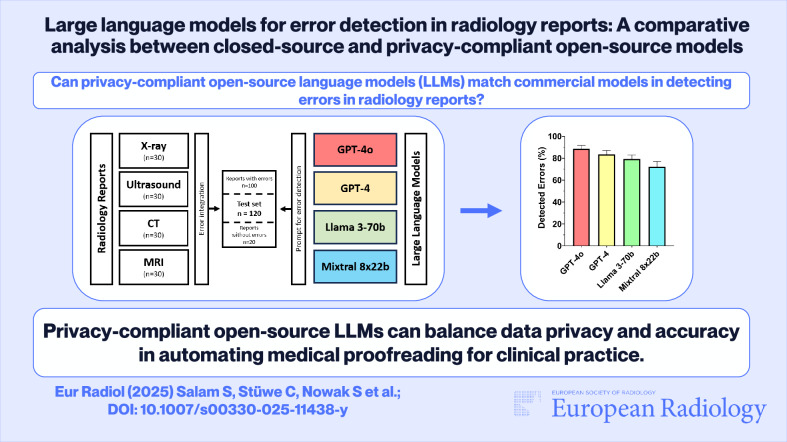

## Introduction

Proofreading radiology reports is an important and time-consuming step in the radiologic workflow. Preliminary reports are often drafted by residents and subsequently reviewed, corrected, and finalized by attending radiologists. This legally mandated process enhances the accuracy of diagnostic reports but is time-consuming and resource-intensive. Moreover, the increasing workload of radiologists [1], high-pressure clinical environments, and unreliable speech recognition systems [2, 3] contribute to errors and discrepancies in radiology reports. Common errors in reports include typographical, numerical and interpretation errors, as well as omissions, insertions and discrepancies between the description of findings and the final impression [2, 4]. If left uncorrected, these errors can have negative consequences for patients [3, 4]. Currently, tools for correcting these errors that go beyond spelling and grammar checking are not routinely available.

Recent studies have highlighted potential applications of large language models (LLMs) in patient care [5–7]. One of these studies examined the performance of GPT-4 in detecting common errors and discrepancies in radiology reports. The results indicated that GPT-4 could achieve an error detection performance comparable to that of board-certified radiologists, with the potential to significantly reduce work hours and costs [8, 9]. Despite the demonstrated clinical benefits, the use of commercial LLMs like GPT-4 in clinical settings poses significant data privacy challenges. Their cloud-based nature conflicts with the necessity for data confidentiality, which currently makes them unsuitable for handling real-world medical data in clinical practice [10]. Consequently, there is a need for privacy-compliant, high-performance LLM models that can be run locally in medical institutions [7, 9]. In this context, the use of open-source LLMs, such as Llama or Mixtral, which can be integrated into local hospital infrastructures, offers a promising alternative.

This study aimed to evaluate and compare the performance of commercially available closed-source and open-source LLMs in detecting common errors in radiology reports.

## Materials and methods

After data privacy consultation, specific approval from the institutional review board was not required under Section 6 (3) of the German Health Data Usage Act, as no patient-identifying information was provided to the LLMs.

### Preparation of radiology report dataset

**Fig. 1:**
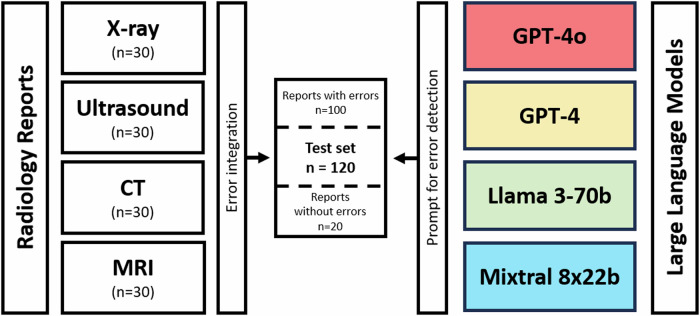
Study flowchart. A total of 120 radiology reports were compiled, with 30 reports from each modality (X-ray, ultrasound, CT, and MRI). Errors were then intentionally inserted into 100 of these reports, while 20 reports remained unchanged. Two commercial closed-source (GPT-4, GPT-4o) and two open-source models (Llama 3-70b, Mixtral 8x22b) were tasked with detecting errors using identical prompts

A total of 120 radiology reports (30 each from X-ray, ultrasound, CT, and MRI reports; mean words per report: 198 ± 86) were generated by different board-certified radiologists (A.I., D.K., D.L.K., J.A.L.) based on real reports from the institutional database. These included internal and external hospital reports, as well as outpatient clinics, covering a broad spectrum of report styles and pathologic abnormalities. No sensitive personal information from existing reports was used. The dataset was divided into two groups: reports with integrated errors (*n* = 100) and reports without integrated errors (*n* = 20). Within the first report set, a radiology resident (B.S.) intentionally inserted a various number of errors with a maximum of seven errors per report. The focus during error insertion was to replicate realistic clinical scenarios, with some reports containing only a few errors while others included several. Following error insertion, the modified reports were re-evaluated by the aforementioned board-certified radiologists. Errors were either retained or removed based on a consensus decision. Ultimately, a total of 397 errors (median: 4, minimum: 2, maximum: 7) were included in the final set of reports. The following five error categories were defined to encompass the most common types of errors in radiology reports: (1) Typographical errors (e.g., transposed letters) or words that might be misrecognized by speech recognition systems due to similar prefixes; (2) Numerical errors in the report, including transposed numbers, comma errors, calculation mistakes, and incorrect units of measurement; (3) Discrepancies between the findings and the impression (e.g., differing localization or incorrect anatomical structure naming); (4) Missing or added words in the impression that alter the meaning; (5) Interpretation errors, such as poor communication-related errors, under-reading errors, or misidentification of findings with incorrect causation [2, 4, 9]. Only the errors intentionally introduced into the text were used as the reference standard. To ensure that no additional errors were present in the radiology report texts, they were reviewed by three readers (B.S., D.K., and A.I., with 3, 6, and 7 years of experience in radiology, respectively). Discrepancies were resolved through a consensus reading among all three readers. The study design is summarized in Fig. 1.

### Error detection

**Fig. 2:**
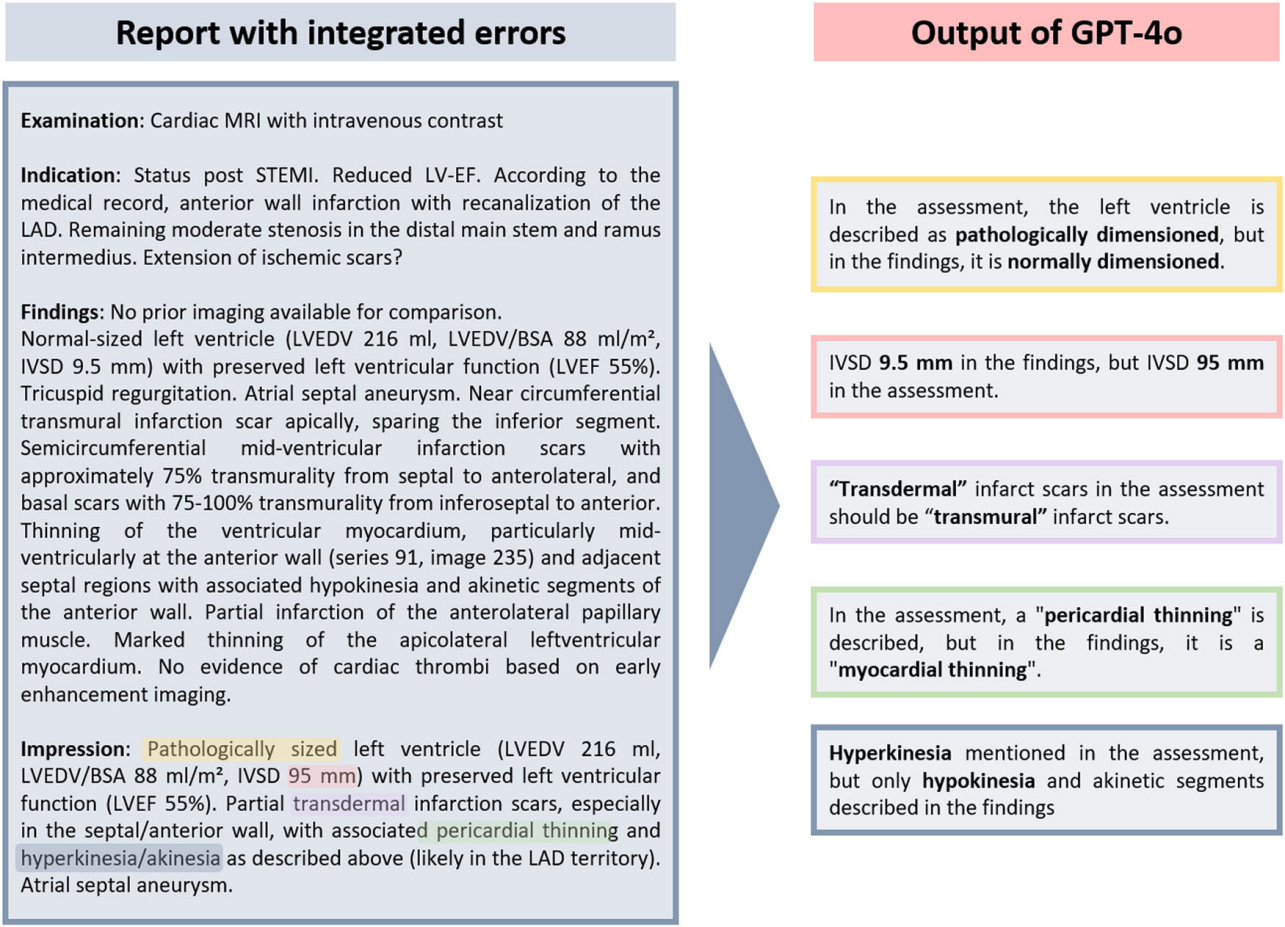
Example of a radiology report with highlighted errors, along with the corresponding correction output generated by GPT-4o

Two non-quantized open-source LLMs, Llama 3-70b (version: June 2024; Meta) and Mixtral 8x22b (version: June 2024; Mistral AI) and two commercial models, GPT-4 and its successor GPT-4o (version: June 2024; OpenAI), were used to evaluate the radiology reports for errors. The commercial GPT models were assessed via OpenAI’s web-based interface (https://chatgpt.com/), and the open-source models were applied using a web-based interface provided by the University of California, Berkeley (https://lmarena.ai/) [11]. The LLMs were tasked with identifying errors in each radiology report using zero-shot prompting [9, 12]. The final prompt was as follows: “You are a radiology specialist. Your task is to review radiology reports for errors and correct them, paying particular attention to the following error types: (1) Typographical errors (e.g., transposed letters) or words that might be misrecognized by speech recognition systems due to similar prefixes. (2) Numerical errors in the report, including transposed numbers, comma errors, calculation mistakes, and incorrect units of measurement. (3) Discrepancies between findings and impression (e.g., differing localization or incorrect anatomical structure naming). (4) Missing or added words in the impression that alter the meaning. (5) Interpretation errors, including poor communication-related errors, under-reading errors, or misidentification of findings with incorrect causation. (6) Other definitive professional report errors. List all identified errors briefly and succinctly in a table.” The prompt was derived heuristically on several fictious radiology reports. Among different prompt designs, this prompt was perceived to robustly detect most errors. No reports from the validation dataset were used during this phase. Only zero-shot prompt approach was used. The time required for the LLMs to proofread each radiology report was measured (from the submission of the prompt to the receipt of the complete response). An exemplary report with highlighted errors, along with the corresponding correction output from GPT-4o, is provided in Fig. 2.

### Statistical analysis

All statistical analyses were conducted using IBM SPSS Statistics version 29.0 (IBM) and Prism version 10.3.0 (GraphPad). The number of correctly detected errors and the time taken to process each radiology report were evaluated as outcome measures. The number of errors detected correctly by the different LLMs was compared using Kruskall–Wallis tests. The Wilson method was employed to calculate 95% confidence intervals (CIs). This analysis was performed for all modalities and error categories combined and separately for each LLM. The average processing time of the LLMs was compared using paired-sample *t*-tests. The Bonferroni correction was applied to adjust for multiple comparisons. A two-sided *p*-value < 0.05 was considered statistically significant.

## Results

### Comparison of processing time between LLMs

The LLMs took an average of 10 ± 5 s (range: 4–21 s) to process the error detection. The open-source models Llama-70b and Mixtral 8x22b were significantly faster than the closed-source LLMs GPT-4 and GPT-4o (6 ± 2 s vs. 13 ± 4 s; *p* < 0.001). In the direct comparison, Llama-70b had the shortest processing time, followed by Mixtral 8x22b, GPT-4, and GPT-4o (5 ± 1 s vs. 7 ± 2 s vs. 12 ± 3 s vs. 15 ± 4 s; *p* < 0.001).

### Overall performance in detecting errors

**Fig. 3:**
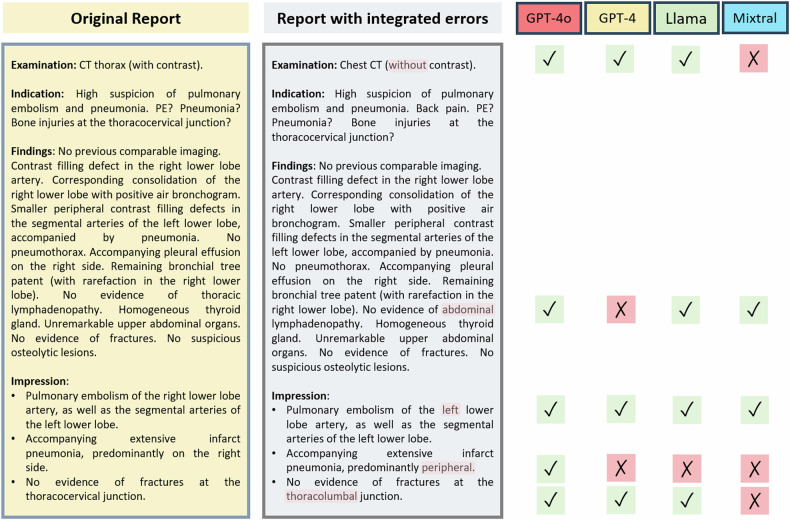
Proofreading example with different detection performance of GPT-4, GPT-4o, Llama 3-70b, and Mixtral 8x22b. Integrated errors are highlighted in red in the text. For each model, correct detection of the error is marked with a green tick, non-detection of an error with a red cross

**Fig. 4:**
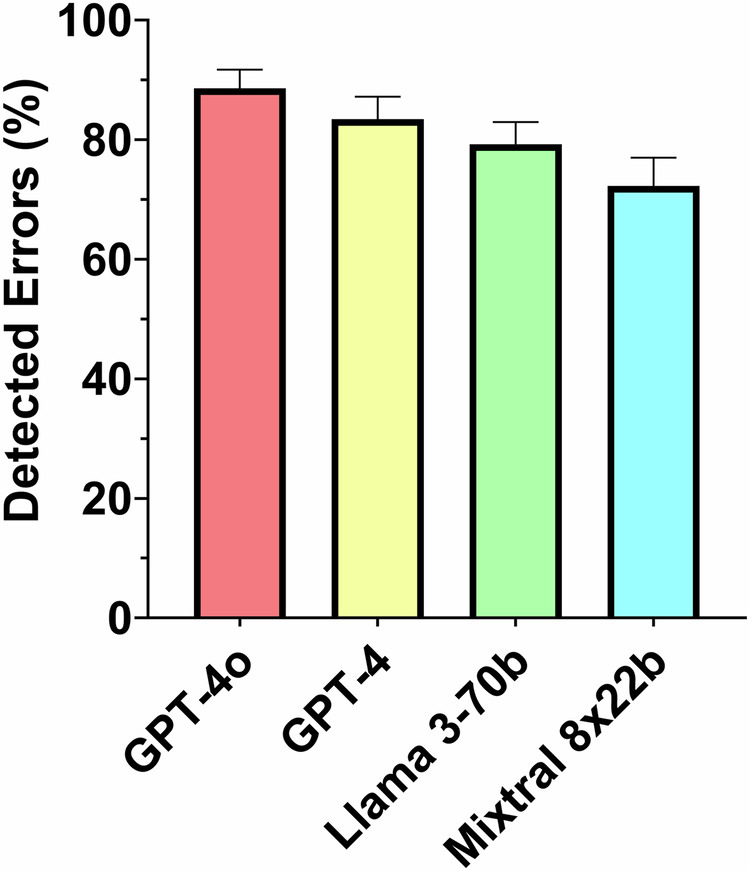
Bar graph shows the percentage of detected errors for GPT-4o, GPT-4, Llama 3-70b, and Mixtral 8x22b. The error bars are 95% CIs

GPT-4o achieved the highest error detection rate (88% [348 of 397; 95% CI: 86, 92]), followed by GPT-4 (83% [328 of 397; 95% CI: 80, 87]), Llama 3-70b (79% [311 of 397; 95% CI: 76, 83]), and Mixtral 8x22b (73% [288 of 397; 95% CI: 68, 77]; *p* < 0.001) (Figs. 3 and 4). There was no statistical difference found between GPT-4o and GPT-4 (*p* = 0.34) or between Llama 3-70b and Mixtral 8x22b (*p* = 0.26). A significant difference was found between GPT-4 and Mixtral 8x22b (*p* = 0.001), but not between GPT-4 and Llama 3-70b (*p* = 0.50). Overall, the closed-source LLMs demonstrated a higher error detection rate than the open-source LLMs (85% [676 of 794; 95% CI: 84, 88] vs. 75% [599 of 794; 95% CI: 73, 79]; *p* < 0.001). Further results are provided in Table 1.

**Table 1:** Comparison of error detection rates between commercially available (GPT-4, GPT-4o) and open-source LLMs (Llama 3-70b, Mixtral 8x22b) in identifying common errors in radiology reports

	GPT-4 (%)	GPT-4o (%)	Llama 3-70b (%)	Mixtral 8x22b (%)	*p*-value
CT	83 [76, 91]	91 [86, 96]^║^	82 [74, 89]	68 [58, 79]^‡^	0.004
MRI	77 [68, 86]	82 [75, 90]	77 [70, 84]	70 [60, 79]	0.211
X-Ray	88 [80, 94]^§^	93 [88, 99]^§║^	72 [64, 81]^†‡^	75 [65, 86]^‡^	< 0.001
Ultrasound	86 [79, 93]	87 [81, 95]	86 [79, 93]	76 [67, 84]	0.093
Total	83 [80, 87]^║^	88 [86, 92]^§║^	79 [76, 83]^†^	73 [68, 77]^†‡^	< 0.001

Mean is given with 95% confidence interval in squared parenthesis

Bonferroni correction for multiple comparisons

^†^ *p*-value < 0.05 against GPT4o

^‡^ *p*-value < 0.05 against GPT4

^§^ *p*-value < 0.05 against Llama 3-70b

^║^ *p*-value < 0.05 against Mixtral 8x22b

### Performance in detecting errors by imaging modality

Overall across all LLMs, no significant differences were observed in error detection between the four imaging modalities (X-ray: 81% [277 of 340; 95% CI: 78, 86] vs. ultrasound: 83% [299 of 360; 95% CI: 83, 79] vs. CT: 81% [366 of 452; 95% CI: 77, 85] vs. MRI: 76% [333 of 436; 95% CI: 73, 80]; *p* = 0.32). Subgroup analysis revealed significant differences between the closed-source and open-source LLMs for error detection in X-ray reports (90% [153 of 170; 95% CI: 86, 95] vs. 74% [124 of 170; 95% CI: 67, 81]; *p* < 0.001) and in CT/MRI reports (83% [369 of 444; 95% CI: 80, 87] vs. 74% [330 of 444; 95% CI: 70, 78]; *p* < 0.001). No significant differences were observed between the closed-source and open-source LLMs in the detection of errors in ultrasound reports (87% [156 of 180; 95% CI: 82, 92] vs. 81% [145 of 180; 95% CI: 75, 86]; *p* = 0.10). In the direct comparison of individual LLMs, significant differences were found in error detection for CT and MRI reports between GPT-4o, GPT-4, Llama 3-70b and Mixtral 8x22b (86% [191 of 222; 95% CI: 82, 91] vs. 79% [178 of 222; 95% CI: 74, 86] vs. 79 [175 of 222; 95% CI: 74, 84] vs. 70% [155 of 222; 95% CI: 62, 76]; *p* < 0.001). Here, no significant differences were found when comparing the best-performing closed-source (GPT-4o) with the best-performing open-source LLM (Llama 3-70b) (*p* = 0.09). Additional results are presented in Table 1.

### Performance in detecting errors by error type

Numerical errors (88% [67 of 76; 95% CI: 82, 93]) were detected significantly more often than typographical errors (75% [65 of 86; 95% CI: 68, 82]; *p* = 0.02), discrepancies between findings and impression (73% [73 of 101; 95% CI: 67, 80]; *p* = 0.003), and interpretation errors (70% [50 of 71; 95% CI: 62, 78]; *p* = 0.001). There were no significant differences in error detection among other error categories. Further results are displayed in Table 2.

**Table 2:** Comparison of error detection rates for different error types in radiology reports (typographical errors, numerical errors, discrepancies between the findings and the impression, omission or insertion of words, interpretation errors) between commercially available (GPT-4, GPT-4o) and open-source LLMs (Llama 3-70b, Mixtral 8x22b)

	Typographical (%)	Numerical (%)	Findings/impression (%)	Omission/insertion (%)	Interpretation (%)	*p*-value
GPT-4	83 [74, 91]	91 [84, 98]^#^	82 [75, 91]	81 [71, 91]	72 [60, 83]^‡^	0.030
GPT-4o	83 [75, 92]	95 [90, 99]^§^	76 [66, 86]^‡^	93 [86, 99]	78 [66, 89]	0.004
Llama	66 [55, 77]^‡║^	88 [80, 96]^†#^	75 [65, 85]	85 [77, 95]	65 [52, 78]^‡^	0.001
Mixtral	67 [56, 78]	76 [65, 86]	62 [50, 73]	75 [63, 86]	67 [54, 80]	0.351
Total	75 [68, 82]^‡^	88 [82, 93]^†§#^	73 [67, 80]^‡^	83 [77, 90]	70 [62, 78]^‡^	< 0.001

Mean is given with 95% confidence interval in squared parenthesis

Bonferroni correction for multiple comparisons

^†^ *p*-value < 0.05 against typographical errors

^‡^ *p*-value < 0.05 against numerical errors

^§^ *p*-value < 0.05 against findings-impression discrepancies

^║^ *p*-value < 0.05 against omission/insertion errors

^#^ *p*-value < 0.05 against interpretation errors

### Qualitative analysis of false positives in error- and error-free reports

When analyzing the 100 modified reports, there were no cases of false positive errors detected (0%). In detecting errors within the 20 error-free reports, across all LLMs, only one instance of a false positive was identified in the 80 error analyses (1.25%). In this case, Llama 3-70b incorrectly marked the phrase “Interim extension of the dorsal spondylodesis cranially, currently from T7 to S1” in a spine X-ray report as an “expert interpretation error,” suggesting it should read L1 instead of S1. This might presumably be explained by the examination description “thoracolumbar spine” (without specific mention of the sacral spine). The other LLMs did not flag this as an error. There were several instances where the LLMs provided suggestions for improvement rather than marking errors. For example, GPT-4o suggested in a groin ultrasound report that instead of “hernial orifice measuring up to 2 cm,” the size of the orifice should be provided in additional planes. In another case, Mixtral 8x22b suggested enhancing clarity in a CT abdomen report by converting “kidney stone measuring approximately 9 × 13 mm” to “kidney stone measuring approximately 0.9 × 1.3 cm,” as the rest of the report consistently used centimeters. These suggestions were clearly marked as recommendations for improvement and were not considered false positive errors.

## Discussion

The present study evaluated privacy-compliant open-source LLMs for error detection in radiology reports and compared their performance and processing time with that of established commercial closed-source LLMs. Our findings indicate that the open-source models Llama 3-70b and Mixtral 8x22b demonstrate good overall error detection capabilities, although their performance is lower than that of commercial models like GPT-4o and GPT-4, particularly in identifying numerical discrepancies and inconsistencies between findings and impressions.

Gertz et al demonstrated that GPT-4 can achieve error detection comparable to board-certified radiologists, potentially leading to a significant reduction in work hours and costs [9]. Our study not only confirms the strong error detection capabilities of GPT-4 but also highlights the superior performance of GPT-4o compared to its predecessor. Although open-source models showed good performance in our study, they were inferior to that of the evaluated closed-source LLMs. The superior performance of commercial closed-source LLMs, especially GPT-4o, may reflect the benefits of extensive data training and refinement; however, the underlying factors contributing to this performance remain uncertain.

However, since GPT-4o and other closed-source LLMs are cloud-based, their use involves external data processing [10]. This raises potential privacy concerns, particularly for sensitive information, and poses risks regarding data confidentiality and compliance with strict regulations governing medical data handling [13]. This issue is particularly relevant in clinical settings, where the protection of patient data is of utmost importance. In contrast, open-source models offer a substantial advantage in terms of data privacy, as they can be integrated into local hospital infrastructures, ensuring that sensitive patient data remains on-site. This setup significantly reduces the risk of data breaches and unauthorized access [14]. The use of locally hosted open-source LLMs aligns with legal, ethical, and data security standards in healthcare, making the use of such models feasible in real clinical practice. A recent study evaluated the capability of a local open-source language model in extracting medical information from real-world free-text radiology reports, demonstrating excellent accuracy without the need for specific training [15]. In another publication, Adams et al compared the performance of open-source LLMs with leading closed-source models in answering questions from the American College of Radiology In-Training Examination [16]. The use of Llama 3 achieved comparable results to leading closed-source LLMs, including OpenAI’s GPT-4. Similarly, in our study, open-source models, particularly Llama 3-70b, demonstrated results comparable to those of closed-source models. The increasing maturity and competitiveness of open-source models make them promising candidates for future research and applications in radiology.

Regarding time savings, our findings are consistent with other studies examining the integration of LLMs into radiologic workflows [5, 9, 17]. When used as a proofreading tool, LLMs with an average generation time of only 10 s (as demonstrated in our findings), could be more cost-effective compared to human readers. Our results show that the open-source LLMs were significantly faster than the GPT-4 models. Although not published by OpenAI, it is estimated that GPT-4 has over 1000 trillion parameters [18]. Therefore, we speculate that this difference in speed may be attributed to substantial differences in model size between the open-source LLMs and GPT-4. Although we lack detailed information on the GPU memory requirements of the open-source LLMs and their associated costs, it is reasonable to assume that fine-tuning a smaller open-source model could improve performance, potentially narrowing the gap with commercial tools while significantly reducing costs compared to radiologist-led error detection.

We observed a variance in LLM performance across imaging modalities, with significant differences between commercial closed-source and open-source models in detecting errors in X-ray and CT/MRI reports. This highlights the potential of commercial models to process complex and diverse radiological data while also indicating that open-source models need further development to enhance their accuracy across various imaging modalities.

Our results further demonstrate that certain error types, such as numerical errors, are detected more accurately than others, such as interpretative errors or discrepancies between findings and impressions. These differences may be partly attributed to the nature of the task: whereas radiologists in clinical practice have access to both written reports and imaging data, LLMs in this study relied solely on textual information, which may not always clearly reveal certain error types. Due to these limitations and legal obligations, LLM-assisted error detection should not be seen as a replacement for human review but rather as a complementary tool to identify major inconsistencies in written reports. This capability could be particularly useful in clinical practice, not only for attending radiologists but also for junior doctors in performing initial error checks, thereby streamlining and enhancing the correction process. Moreover, previous research suggests that human performance may decline due to demands of multitasking and off-hours shifts, whereas LLMs may maintain consistent performance [9, 19, 20].

Despite the promise shown by open-source LLMs as privacy-compliant alternatives, their lower detection rates, particularly for critical errors, highlight the need for ongoing refinement and domain-specific training to enhance their accuracy. This is especially crucial in the context of clinical decision-making, where even minor errors can significantly impact patient outcomes [3, 4].

Our study has several limitations. First, the controlled nature of our experimental setup, with manually generated predefined errors introduced into a selected set of radiology reports, cannot fully capture the variability and complexity of errors encountered in routine clinical practice. This limitation could affect the generalizability of our findings, as real-world errors are often more nuanced and context-dependent. While the tested models demonstrated good performance in this controlled environment, their performance in real-world settings, where they might encounter unfamiliar or differently structured input, remains to be evaluated. In our study, we applied LLMs via web interfaces that did not allow setting fixed random seeds, and with temperatures greater than 0. Due to the autoregressive generative nature of LLMs, results would differ with identical inputs and thus are not exactly reproducible. Further, we did not explore instructing LLMs to generate responses in structured, machine-readable JavaScript Object Notation (JSON). This approach would facilitate the structured storage of results in databases. Instead, we focused on evaluating direct listings of sections with errors by LLMs, believing this more human-readable format would be more practical for physicians aiming to proofread reports post-creation. Due to the scope of our study design, the potential impact of domain-specific fine-tuning on the performance of LLMs and their integration into existing workflows remains unclear. Also, it is uncertain to what extent performance improvements can be achieved through targeted prompt optimization. Additionally, only zero-shot prompt approach was used in our study. Given the differences in model size, open-source models could benefit from few-shot prompts, potentially narrowing the performance gap with commercial LLMs. These limitations underscore the need for further studies to explore how immediate error feedback from LLMs in a clinical setting might enhance the overall quality of radiology report findings.

In conclusion, the potential of open-source LLMs to provide a privacy-compliant solution for error detection in radiology reports is substantial. The integration of locally operated LLMs into clinical practice could enable automated proofreading without compromising data privacy laws. While further improvements of these models should focus on accuracy for medical and radiological data, the development of privacy-compliant, high-performance open-source LLMs would create a powerful tool advancing radiology reporting, and lead to more accurate, timely diagnoses as well as better patient care.

## Supplementary information

ELECTRONIC SUPPLEMENTARY MATERIAL

## Abbreviations

GPT-4: Generative pre-trained transformer 4
LLMs: Large language models
