# Supplementary material for: Large language models for error detection in radiology reports: a comparative analysis between closed-source and privacy-compliant open-source models
Source: Eur Radiol. 2025 Feb 20;35(8):4549–57. doi: 10.1007/s00330-025-11438-y (PMC12226608; doi:10.1007/s00330-025-11438-y)
Supplement: Supplementary file 1 — ELECTRONIC SUPPLEMENTARY MATERIAL [file 330_2025_11438_MOESM1_ESM.docx]

**Supplemental Table 1.** Comprehensive table comparing the number of detected errors for each type (Error type 1-5) and modality (CT, MRI, X-Ray, Ultrasound) between commercially available (GPT-4, GPT-4o) and open-source LLMs (Llama 3-70b, Mixtral 8x22b).

|  |  | Total Error Count –  CT | Total Error Count –  MRI | Total Error Count –  X-Ray | Total Error Count –  Ultrasound | Total Error Count – All modalities |
| --- | --- | --- | --- | --- | --- | --- |
| Error type 1 | **GPT-4** | 21 (78 %) | 20 (80 %) | 17 (89 %) | 13 (87 %) | 71 (83 %) |
|  | **GPT-4o** | 23 (85 %) | 19 (76 %) | 17 (89 %) | 13 (87 %) | 72 (84 %) |
|  | **Llama** | 20 (74 %) | 14 (56 %) | 8 (42 %) | 12 (80 %) | 54 (63 %) |
|  | **Mixtral** | 19 (70 %) | 18 (72 %) | 13 (68 %) | 9 (60 %) | 59 (69 %) |
|  |  | | | | | |
| Error type 2 | **GPT-4** | 19 (100 %) | 19 (86 %) | 18 (100 %) | 16 (94 %) | 72 (95 %) |
|  | **GPT-4o** | 19 (100 %) | 22 (100 %) | 17 (94 %) | 16 (94 %) | 74 (97 %) |
|  | **Llama** | 18 (95 %) | 19 (86 %) | 14 (78 %) | 17 (100 %) | 68 (89 %) |
|  | **Mixtral** | 15 (79 %) | 17 (77 %) | 14 (78 %) | 15 (88 %) | 61 (80 %) |
|  |  | | | | | |
| Error type 3 | **GPT-4** | 29 (91 %) | 19 (83 %) | 13 (81 %) | 22 (73 %) | 83 (82 %) |
|  | **GPT-4o** | 28 (88 %) | 18 (78 %) | 16 (100 %) | 22 (73 %) | 84 (83 %) |
|  | **Llama** | 26 (81 %) | 19 (83 %) | 14 (88 %) | 22 (73 %) | 81 (80 %) |
|  | **Mixtral** | 20 (63 %) | 14 (61 %) | 14 (88 %) | 19 (63 %) | 67 (66 %) |
|  |  | | | | | |
| Error type 4 | **GPT-4** | 16 (84 %) | 11 (69 %) | 13 (87 %) | 11 (85 %) | 51 (81 %) |
|  | **GPT-4o** | 18 (95 %) | 13 (81 %) | 15 (100 %) | 13 (100 %) | 59 (94 %) |
|  | **Llama** | 16 (84 %) | 14 (88 %) | 13 (87 %) | 13 (100 %) | 56 (89 %) |
|  | **Mixtral** | 15 (79 %) | 11 (69 %) | 11 (73 %) | 11 (85 %) | 48 (76 %) |
|  |  | | | | | |
| Error type 5 | **GPT-4** | 9 (56 %) | 15 (65 %) | 13 (76 %) | 14 (93 %) | 51 (72 %) |
|  | **GPT-4o** | 14 (88 %) | 17 (74 %) | 14 (82 %) | 14 (93 %) | 59 (83 %) |
|  | **Llama** | 12 (75 %) | 17 (74 %) | 11 (65 %) | 12 (80 %) | 52 (73 %) |
|  | **Mixtral** | 9 (56 %) | 17 (74 %) | 12 (71 %) | 15 (100 %) | 53 (75 %) |
